# Prevalence of depression and associated factors among adult cancer patients receiving chemotherapy during the era of COVID-19 in Ethiopia. Hospital-based cross-sectional study

**DOI:** 10.1371/journal.pone.0270293

**Published:** 2022-06-24

**Authors:** Abebe Muche Belete, Anmut Alemagegn, Anemut Tilahun Mulu, Taklo Simeneh Yazie, Bekalu Bewket, Adisu Asefa, Wendimeneh Shibabaw Shiferaw

**Affiliations:** 1 Department of Biomedical Science, College of Health Science, Debre Berhan University, Debre Berhan, Ethiopia; 2 Oncology Clinic, Tikur Anbesa Specialized Hospital, Addis Ababa University, Addis Ababa, Ethiopia; 3 Department of Biomedical Science, College of Health Science, Debre Tabor University, Debre Tabor, Ethiopia; 4 Pharmacology and Toxicology Unit, Department of Pharmacy, College of Health Sciences, Debre Tabor University, Debre Tabor, Ethiopia; 5 Department of Nursing, College of Health Science, Injibara University, Injibara, Ethiopia; 6 Department of Nursing, College of Health Science, Debre Berhan University, Debre Berhan, Ethiopia; Government College University Faisalabad, Pakistan, PAKISTAN

## Abstract

**Background:**

Depression is a major public health problem among cancer patients undergoing chemotherapy. It compromises patient outcomes, resulting in higher rates of mortality. Currently, there are little data on the prevalence of depression in Sub-Sharan countries. Therefore, the current study was done to assess the prevalence of depression among adult cancer patients and its associated factors in Ethiopia.

**Method:**

An institutional based cross-sectional study was conducted among 420 adult cancer patients from 1^st^ March to April 30, 2021. Systematic random sampling technique was used to recruit participants. Depression was assessed using the Patient Health Questionnaire-9. The collected data were coded and entered into Epi-data version 4.2 and exported to SPSS version 25 for analysis. Bivariate binary logistic regression was performed to select factors that will be included in multivariate analysis, and variables with a p-value < 0.05 were included in multivariate analysis. In multivariate analysis, odds ratios and their 95% confidence intervals were computed and variables with p-value < 0.05 were considered to declare a significant association.

**Results:**

In this study, the prevalence of depression was 33.1% (95% CI = 0.2858, 0.3761). Minimal symptoms of depression, minor depression, moderate depression, moderate severe depression, and severe depression were found to be 272 (64.8%), 9 (2.1%), 104 (24.8%), 28 (6.7%), and 7 (1.7%), respectively. Those cancer patients who had unemployed status, sacked from jobs, and had stage IV cancer were most likely to develop depression, whereas patients who completed primary education, colon, prostate and cervical cancer were less likely to have depression.

**Conclusion:**

Depression is found to be a major public health concern for adult cancer patients in Ethiopia. To reduce the occurrence of depression among cancer patients, special attention is needed.

## Background

Worldwide, new cancer cases, and cancer deaths were estimated to be 19.3 million and 10 million, respectively, in 2020. The global cancer burden is expected to be 28.4 million cases in 2040, a 47% rise from 2020 [1]. Well-recognized risk factors for cancer are tobacco use, poor physical activity, poor nutrition [2], obesity [3] and excessive alcohol consumption [4, 5]. The treatment of cancer includes three main modalities namely surgery, radiotherapy and chemotherapy. While the novel strategies may include targeted therapy, electric field treatments and vaccine therapy [6]. Chemotherapy is an intense and cyclic treatment with many side effects such as hair loss, nausea, vomiting and diarrhea [7]. Cyclic dependent kinase inhibitors that have emerged as a potent strategy for the treatment of advanced cancers [8]. The importance of chemotherapy for cure of cancer is increasing, especially with its use as adjuvants to local therapy. Besides, in advanced disease, chemotherapy has an expanding role in efforts to relieve cancer-related symptoms and to prolong life [9].

Cancer remains one of the most feared illnesses and the diagnosis of cancer has a huge psychological impact on the patients and their care-givers [10]. Many cancer patients suffer from psychological problems, such as depression. Cancer patients undergoing chemotherapy, usually experience various symptoms such as depression. Depression worsens during chemotherapy, persists for a long time after the end of chemotherapy, and it is also manifested in the recurrence of the disease [11]. This may interfere with the patient’s ability to cope with the burden of the illness, it may decrease the acceptance of treatment, extend hospitalization [12], reduce the quality of life [13, 14], and increase suicide risk [15]. Furthermore, the depression compromises patient outcomes, resulting in higher rates of mortality [16–18], with estimates as high as a 26% greater mortality rate among patients with depressive symptoms and a 39% higher mortality rate among those with a diagnosis of major depression [19].

The epidemiology of depression among cancer patients was to 23.4% in China [20], 48.7% in Pakistan [21], 17% in Australia [22], 38.2% in Greece [23], 46.5% in Saudi Arabia [24], 67.7% in Rwandan [25], 25% in Addis Ababa-Ethiopia [26], and 58.4% in Gondar-Ethiopia [27]. Different studies showed several factors that are associated with depression such as cancer types [28], female sex [29], old age [14, 30], the duration of cancer [31] and type of treatment. Furthermore, a slightly higher incidence of depression was found among cancer patients who underwent chemotherapy than patients who had not received chemotherapy [32]. Besides, depressive symptoms were more prevalent across patients who were hospitalized compared with patients in the outpatient setting [33].

Regarding depression among cancer patients, most of the studies came from the developed world, and very limited studies addressed populations from low- and middle-income countries. Therefore, this study aimed to assess the prevalence of depression among cancer patients at Tikur Anbessa Specialized Hospital in Ethiopia.

## Methods

### Study area, design and period

The study was conducted at Tikur Anbessa Specialized Hospital (TASH) which is located in Addis Ababa, which is the capital city of Ethiopia. TASH oncology unit was established in 2015 under the Federal Minister of Health. It is located at Addis Ababa, Lideta Sub–City. The oncology unit provides health services for all cancer patients attending TASH from Addis Ababa city, and different regional states of Ethiopia. The oncology unit at TASH is the largest referral site in the country, providing service to more than 60,000 patients annually. It is the sole oncology referral and the only radiotherapy center in Ethiopia. The cross-sectional study design was employed from 1^st^ March to April 30, 2021.

#### Source population

All adult cancer patients who had followed up at TASH and treated with chemotherapy have been taken as the source population.

#### Study population

All adult cancer patients under chemotherapy treatment and follow up service during the data collection period.

### Eligibility criteria

#### Inclusion criteria

All adult cancer patients under chemotherapy and follow up at oncologic clinic in TASH during the data collection period.

#### Exclusion criteria

Patients who had communication or hearing impairments were excluded.

### Sample size calculation and sampling techniques

The required sample size was determined using a single population proportion formula having the following assumptions: value for the 95% CI (Zα/2 = 1.96), the proportion of depression (P = 50%), and margin of error (d = 5%) Then, by adding 10% of study subjects as non-response rate, the final sample size was 422. The study subjects were selected using the systematic random sampling technique after determining the sampling fraction (k = 900/420 = 2) and the first participant was selected using the lottery method.

### Study variables

#### Dependent variable

Depression (Yes/No).

#### Independent variables

Socio-demographic variables: Age, educational status, marital status, employment, and monthly income.

Psychosocial factors: Social and husband support, emotional violence, physical violence, and sexual violence.

Substance use: Use any substance like khat, alcohol, and cigarette.

Clinical characteristics: Stage of cancer, duration of the illness, comorbidity, type of cancer.

Family history of psychiatric illness: A family history (first-degree relatives) of psychiatric problems.

### Data collection tools and procedures

A structured interviewer-administered questionnaire was used to collect data from all participants. Socio-demographic, clinical, and psychosocial factors were assessed using predefined checklists. The social support level was assessed using the Oslo social support scale-3. The Oslo social support-3 consists of three items assessing the level of social support. The sum score ranges from 3 to 14, with high values representing strong levels and low values representing poor levels of social support [34]. Depression was assessed using Patient Health Questionnaire-9 (PHQ-9). The PHQ-9 comprises nine items that can be scored from 0 (not at all) to 3 (nearly every day) and the total score ranges from 0 to 27 to measure depression severity [35, 36]. The standard PHQ9 cut off points 1–4, 5–9, 10–14, 15–19 and greater than or equal to20 was considered as having minimal depression symptoms, minor depression, moderate depression, moderately severe and major depression (severe), respectively. Then, the total depression score was dichotomized, and those cancer patients who scored 10 and above were considered as having symptomatic depression. PHQ-9 is a validated tool in Ethiopia [37].

### Data analysis

The collected data were entered into Epi-data version 4.2 and analyzed by SPSS version 25. Bivariate logistic regression was done and, variables with p-value < 0.05 were entered in to a multivariate logistic model. In multivariate logistic regression analysis, adjusted odds ratio with a p-value of < 0.05 was considered statistically significant.

### Data quality control

The questionnaire was prepared in English and translated to Amharic and translated back to English before the data collection process. The data collection instrument was pre-tested on 5% of the sample to improve the language clarity and appropriateness of the data collection tools. The estimated time required, and necessary amendments were made after the piloting of the questionnaire. The data were collected by four BSc nursing professionals who were trained for one day on the techniques of data collection, purpose of the study, and ethical considerations. The researcher checked the accuracy, completeness and consistency of questionnaires completed by the data collectors to ensure the quality of data and visited the data collectors as many times as possible to check whether he/she collected the data appropriately. The Amharic version of PHQ-9 is validated in Ethiopia.

### Ethical approval and consent to participate

Ethical clearance was obtained from Addis Ababa University, College of Health Sciences, School of Nursing and Midwifery, Research and Ethics Review Committee (IRB protocol: AAU/CHS/NSG/0020). A collaboration letter for data collection was also obtained from TASH. Verbal informed consent was obtained from each participant. The objective of the study and methods of data collection were briefly clarified and explained for each participant, before enrolling any eligible study participants. Then, verbal informed consent was obtained from each participant. As the study does not impose any harm to the participants and from the experience, patients feel their confidentiality is secured more when they give verbal informed consent than written informed consent. To assure confidentiality, a code number was used instead of the participants’ name or identification number.

### Operational definitions

Poor social support: cancer patients who scored 3–8 on the (Oslo-3) social support scale during cancer.

Moderate social support: cancer patients who scored 9–11 on the (Oslo-3) social support scale during cancer.

Strong social support: cancer patients who scored 12–14 on the (Oslo-3) social support scale during cancer.

## Result

### Socio-demographic characteristics of the participants

Four hundred twenty participants were included in the final analysis in this study with a non-response rate of 99.5%. Majority, 243 (57.9%) were female, 220 (52.4%) were in the age between 35 and 51 years and 103 (24.5%) were above the age of 52 years old. The mean and standard deviation age of the participants was 43.6 (SD±12.37) years, over 297 (69%) were married, and 119 (28.3%) completed secondary school education (Table 1).

**Table 1:** Socio-demographic characteristics of study participants among adult cancer patients at the oncology clinic of TASH, Addis Ababa, Ethiopia, 2021.

Variables	Category	Frequency	Percent
**Sex**	Female	243	57.9
	Male	177	42.1
**Age in years**	18–34	97	23.1
	35–51	220	52.4
	≥52	103	24.5
**Educational status**	Illiterate	45	10.7
	Read and write only	77	18.3
	Primary	94	22.4
	Secondary	119	28.3
	College/university	85	20.2
**Marital status**	Single	47	11.2
	Married	290	69
	Divorced	16	3.8
	Widowed/r	67	16
**Occupation**	Private work	106	25.2
	Civil servant	90	21.4
	House wife	116	27.6
	Others	108	25.7
**Income**	Low	60	14.3
	Medium	113	26.9
	High	247	58.8

### Clinical and behavioral characteristics of the study participants

The clinical characteristics of the study participants indicate that; 142 (33.8%) have breast cancer, 120 (28.6%) were on stage 2 cancer, 52 (12.4%) drunk alcohol but now quitted and 33 (7.9%) of participants quitted smoke. Of the total study participants, 6 (1.4%) study participants had a family history of known mental illness and 2(0.5%) had a diagnosis of chronic kidney disease (Table 2).

**Table 2:** Clinical and behavioral characteristics of study participants among adult cancer patients at oncology clinic of TASH, Addis Ababa, Ethiopia, 2021.

Variables	Category	Frequency	Percent
**Stage of cancer**	Stage 1	28	6.7
	Stage 2	99	23.6
	Stage 3	120	28.6
	Stage 4	173	41.6
**Type of cancer**	Breast cancer	156	37.1
	Colon cancer	53	12.6
	Prostate cancer	32	7.6
	NPC	35	8.3
	Cervical cancer	52	12.4
	Lung cancer	33	7.9
	Bladder cancer	12	2.9
	Thyroid cancer	6	1.4
	Hematologic cancer	12	2.9
	Edwing cancer	24	5.7
	Other	5	1.2
**Months since diagnosis**	<6	203	48.3
	7–12	113	26.9
	>12	104	24.8
**Duration since start of chemotherapy**	1–3 months	190	45.2
	4–6 months	110	26.2
	>6 months	120	28.6
**Family history with known mental illness**	Yes	6	1.4
	No	414	98.6
**Hypertension**	Yes	1	0.2
	No	419	99.8
**Diabetes Mellitus**	Yes	1	0.2
	No	419	99.8
**Chronic kidney disease**	Yes	2	0.5
	No	418	99.5
**Cardiovascular disease**	Yes	1	0.2
	No	419	99.8
**Presence of comorbidities**	Yes	17	4
	No	403	96
**Chewing**	Currently chat chewing	1	0.2
	Previously drunker	29	6.9
	Never used	390	92.9
**Alcohol**	Currently drinker	3	0.7
	Previously drunker	52	12.4
	Never drank	365	86.9
**Smoking status**	Currently smoker	1	0.2
	Previously smoker	33	7.9
	Never smoke	386	91.9

Note: presence of comorbidities = include hypertension, diabetes mellitus and kidney disease

Abbreviations: SD = standard deviation, chronic illness =, NPC = Naso pharengyal, other type of cancer include = Kaposi sarcoma, adenocarcinoma, and oropharia cancer

### Psychosocial factors (in the last 6 months) of cancer patients

From the total of participants, 53 (12.6%) responded that their family or close relatives had died and 30 (7.1%) participants responded as having died a spouse, parent, or child during the disease. 125 (29.8%) responded to having major financial crisis. 20(4.8%) responded to being sacked from their job (Table 3).

**Table 3:** Psychosocial factors (in the last 6 months) of adult cancer patients receiving chemotherapy at TASH, Addis Ababa, Ethiopia, 2021.

Variable	Category	Frequency	Percent
**Serious illness or injury during cancer**	Yes	7	1.7
	No	413	98.3
**Close relative serious illness or injury or assault**	Yes	9	2.1
	No	411	97.9
**Died spouse, parent or child**	Yes	30	7.1
	No	390	92.9
**Died family or close relatives**	Yes	53	12.6
	No	367	87.4
	Yes	125	29.8
**Major financial crisis**			
	No	295	70.2
**Sacked from job**	Yes	20	4.8
	No	400	95.2
**Unemployed/not able to work**	Yes	41	9.8
	No	379	90.2
**Separation due to marital difficulty**	Yes	11	2.6
	No	409	97.4
**Broken off a steady relationship**	Yes	11	2.6
	No	409	97.4
**Serious problems with close friend, neighbor /relative**	Yes	19	4.5
	No	401	95.5
**Lost/stolen property which mattered a lot**	Yes	20	4.8
	No	400	95.2
**Any problems with police/court**	Yes	11	2.6
	No	409	97.4
**Trauma by your wife or husband**	Yes	6	1.4
	No	414	98.6
**Forced sexual activity**	Yes	1	0.2
	No	419	99.8

### Social support among cancer patients

**Fig 1:**
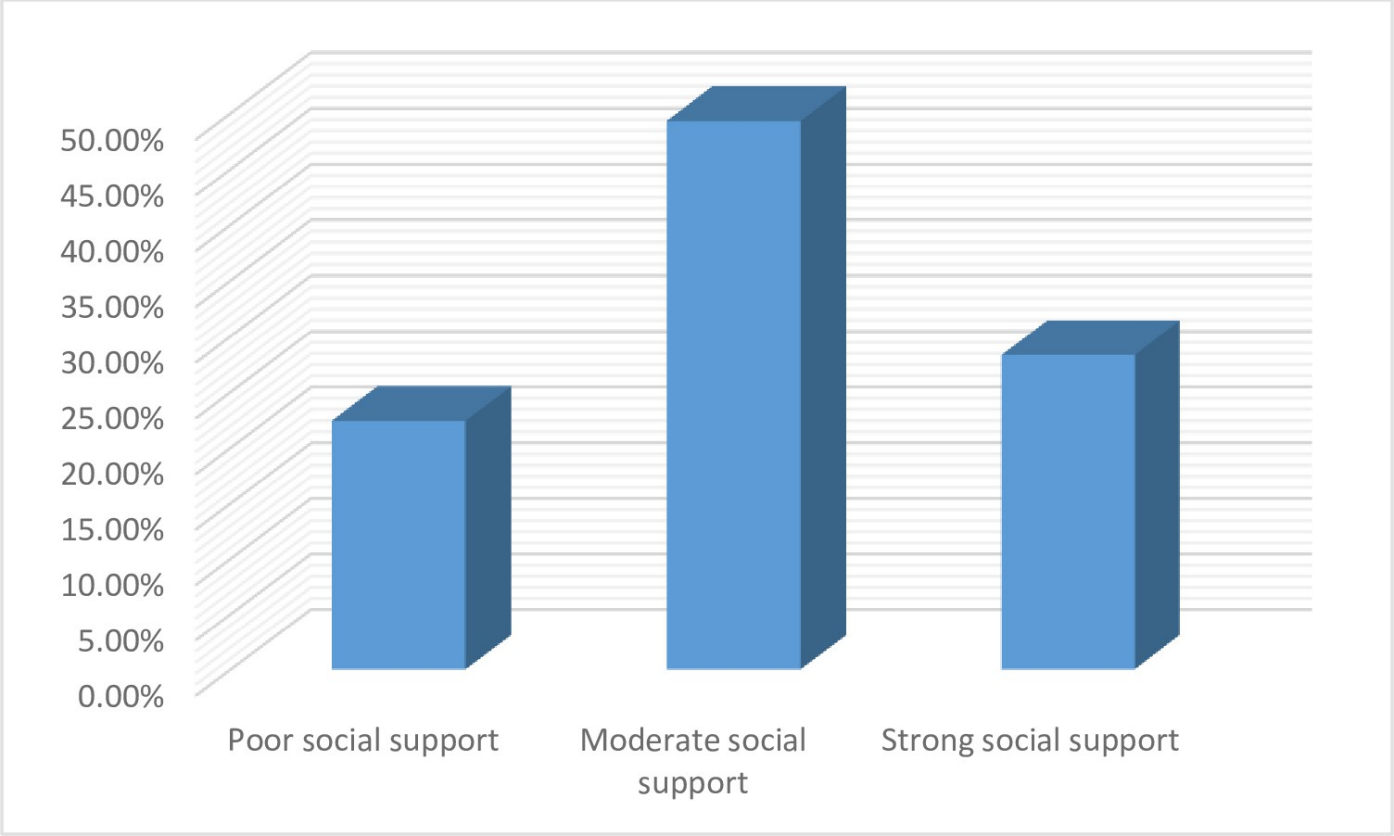
Social support of adult cancer patients.

Of the total participants, 94 (22.4%), 207 (49.3%) and 119 (28.3%) had poor, moderate and strong social support, respectively (Fig 1).

### Prevalence of depression among cancer patients

**Fig 2:**
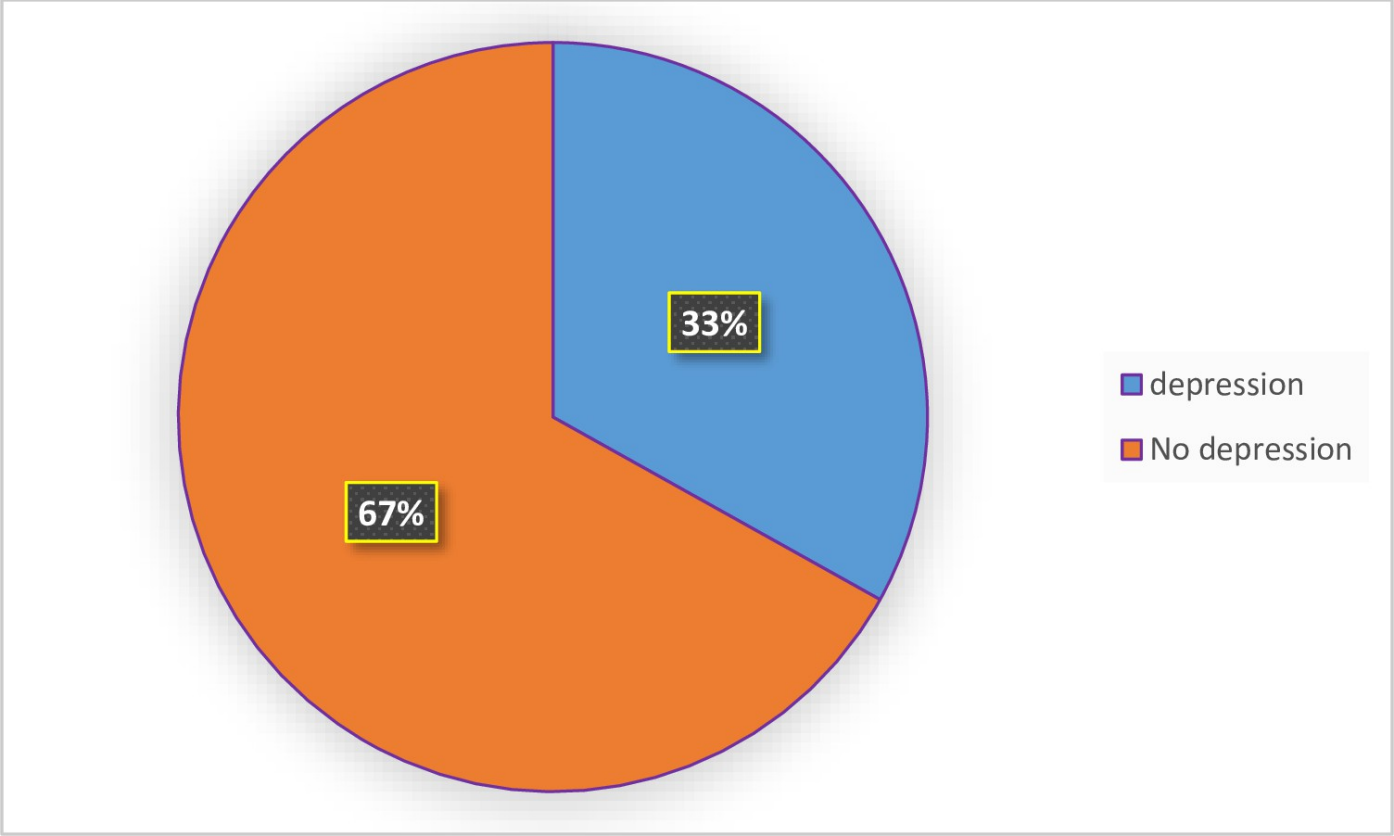
Prevalence of depression among adult cancer patients.

The overall prevalence of depression among adult cancer patients was 33.1% (95% CI = 0.2858, 0.3761) (Fig 2).

**Fig 3:**
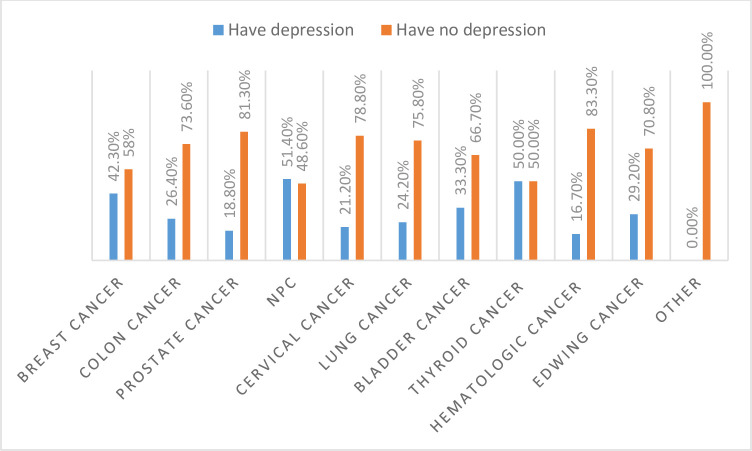
Depression among each type of cancer.

Minimal symptoms of depression, minor depression, moderate depression, moderate severe depression, and severe depression were found to be 272 (64.8%), 9 (2.1%), 104 (24.8%), 28 (6.7%), and 7 (1.7%), respectively. A higher prevalence of depression was seen among patients with nasopharyngeal cancer (NPC) 51%, followed by thyroid cancer 50%, and breast cancer 42% (Fig 3).

### Factors associated to depression

In bivariate logistic regression analysis, education, income, type of cancer, the stage of cancer, serious illness or assault happened to close relatives, major financial problem, unemployed, sacked from a job, a serious problem with a close friend or neighborhood, having some problems with the police or courts, and social support were significantly associated with depression (p <0.05). When these variables were entered and analyzed in multivariate logistic regression, education, colon cancer, prostate cancer, cervical cancer, stage of cancer, unemployed and sacked from job were significantly associated with depression.

The results showed that participants who completed their primary education were less likely to develop depression than those who had no formal education (AOR: 0.414, 95% CI = 0.206–0.829). Additionally, patients with colon cancer were less likely to develop depression as compared to breast cancer (AOR: 0.364, 95% CI = 0.156–0.847). Besides, patients with prostate cancer and cervical cancer were less likely to develop depression compared to patients with breast cancer (AOR: 0.184, 95% CI = 0.064–0.525; AOR: 0.334, 95% CI = 0.148–0.752, respectively). Patients with Stage four cancer were seven times more likely to develop depression compared to patients with stage one (AOR: 7.444, 95% CI = 1.943–28.523). Being unemployed was two times more likely risk of developing depression compared to being employed (AOR: 2.263, 95% CI = 1.043–4.913), and patients sacked from job were three times more likely to develop depression compared to no sacke from job (AOR: 3.404, 95% CI = 1.049–11.042) (Table 4).

**Table 4:** Bivariate and multivariate logistic regression analysis of independent factors to depression among cancer patients at the oncology clinic of TASH, Addis Ababa, Ethiopia, 2021.

Variables	Category	Depression		COR(95%CI)	AOR(95%CI)
		No (n, %)	Yes (n, %)		
**Education**	No formal education	70(57.4)	52(42.)	1	1
	Primary	71(75.5)	23(24.5)	0.436(0.241–0.788)*	0.414(0.206,0.829)*
	Secondary	83(69.7)	36(30.3)	0.584(0.343–0.993)*	0.538(0.279,1.037)
	Higher	57(67.1)	28(32.9)	0.661(0.371–1.178)	0.621(0.291,1.325)
**Income**	Low	32(53.3)	28(46.7)	1.827(1.03,3.239)*	1.211(0.573,2.56)
	Medium	82(72.6)	31(27.4)	0.789(0.483–1.29)	0.791(0.425,1.473)
	High	167(67.6)	80(32.4)	1	1
**Type of cancer**	Breast cancer	90(57.7)	66(42.3)	1	1
	Colon cancer	39(73.6)	14(26.4)	0.49(0.246,0.974)*	0.364(0.156,0.847)*
	Prostate cancer	26(81.3)	6(18.8)	0.315(0.123,0.808)*	0.184(0.064,0.525)*
	NPC	17(48.6)	18(51.4)	1.444(0.692,3.011)	1.408(0.609,3.256)
	Cervical cancer	41(78.8)	11(21.2)	0.366(0.175,0.765)*	0.334(0.148,0.752)*
	Lung cancer	25(75.8)	8(24.2)	0.436(0.185,1.028)	0.407(0.154,1.076)
	Bladder cancer	8(66.7)	4(33.3)	0.682(0.197,2.36)	0.775(0.196,3.065)
	Thyroid cancer	3(50)	3(50)	1.364(0.267,6.97)	1.291(0.19,8.791)
	Hematologic	10(83.3)	2(16.7)	0.273(0.058,1.286)	0.559(0.098,3.184)
	Edwing cancer	17(70.8)	7(29.2)	0.561(0.22,1.431)	0.617(0.215,1.766)
	Other	5(100)		-	-
**Stage of cancer**	Stage 1	25(89.3)	3(10.7)	1	1
	Stage 2	81(81.8)	18(19.2)	1.852(0.504,6.808)	1.893(0.461,7.764)
	Stage 3	85(70.8)	35(29.2)	3.431(0.973,12.104)	3.558(0.904,14.009)
	Stage 4	90(52)	83(48)	7.685(2.237,26.402)*	7.444(1.943,28.523)*
**Serious illness, injury or assault happened to a close relatives**	Yes	35(51.5)	33(48.5)	2.188(1.291,3.707)*	1.9(0.958,3.771)
	No	246(69.9)	106(30.1)	1	1
**A major financial crisis**	Yes	80(56.7)	61(43.3)	0.509(0.333,0.777)*	1.597(0.965,2.642)
	No	201(72)	78(28)	1	1
**Unemployed**	Yes	16(39)	25(61)	3.632(1.868,7061)*	2.263(1.043,4.913)*
	No	165(69.9)	114(30.1)	1	1
**Sacked from job**	Yes	5(27.8)	13(72.2)	5.695(1.988,16.319)*	3.404(1.049,11.042)*
	No	276(68.7)	126(31.3)	1	1
**A serious problem with a close friend, neighborhood**	Yes	7(36.8)	12(63.2)	3.699(1.422, 9.617)*	3.177(0.872,11.571)
	No	274(68.3)	127(31.7)	1	1
**Have you had any problems with the police or courts**	Yes	4(36.4)	7(63.6)	3.672(1.057,12.765)*	0.924(0.164,5.199)
	No	277(67.7)	132(32.3)	1	1
**Social support**	Poor	91(56.9)	69(43.1)	1.896(1.106,3.248)*	1.641(0.851,3.165)
	Moderate	120(74.1)	42(25.9)	0.875(0.499,1.535)	1.196(0.622,2.3)
	Strong	70(71.4)	28(28.6)	1	1

NPC: Naso Pharyngeal Cancer; N: number; COR = Crud Odds ratio; AOR = Adjusted Odds Ratio; CI = Confidence Interval; %: percent

* = statistically significant by univariate logistic regression

** = statistically significant by multivariate logistic regression

## Discussion

The overall prevalence of depression among adult cancer patients was 33.1% in this study. This finding was higher than the results of a studies conducted in Jordan-23.4% [38], India-22% [39], Iran-21.3% [31], Asia-29.6% [40], German-24% [41] and Ethiopia-Addis Ababa-25.0% [26]. However, it was lower than the findings of a study conducted in China- 66.7% [42], Ethiopia-Gondar-70.86% [43], Greece-38.2% [23], German 36.9% [44], Ethiopia 36.9% [45], and Rwanda 67.7% [46]. The variation might be due to differences in instruments to assess depression, criteria to define depression, and included cancer populations with respect to cancer type, stage and treatment modality.

Available data also suggest that cancer may increase patients’ susceptibility to depression in several ways. To mention some, a reaction to a severe diagnosis and the forthcoming deterioration of health status may constitute a risk factor for depression; treatment with immune response modifiers and chemotherapy regimens, and experiencing of metabolic and endocrine alterations, chronic pain and extensive surgical interventions, may represent additional contributing factors [47, 48].

Patients who are at a high risk of depression require special attention and a strong support system. psychological problems hinder their ability to cope with treatment and symptoms, as well as recovery from chemotherapy side effects and cancer impact [49]. Depression in patients with cancer can be prevented and prophylactic treatment should be administered during oncological treatment [50]. The treatment of depression must be started at an early stage. It can take a long time to make patients aware of the psychological nature of their difficulties, having this consideration, encourage them to request a psychological consultation or to accept the prescription of psychotropic or, in particular, antidepressant medicaments [51].

The current study found several associated factors for depression among cancer patients in Ethiopia. Those associated factors were primary education, unemployment, sacked from jobs, colon cancer, prostate cancer, cervical cancer and cancer stage. Study participants who completed their primary education were less likely to develop depression than who had no formal education. This finding was in a line with the findings of studies conducted in Ethiopia [21, 26, 43]. Education may offer opportunities for developing interventions to reduce the disease burden of depression. Low education levels are linked with depression and that higher levels of education play a preventive role against depression [21]. Being unemployed was found to have 2.2 times a more likely risk to develop depression compared to being employed, which is supported by other studies [43, 52]. Patients sacked from job were 3.4 times more likely to develop depression compared to no sack from job. Having no or low education level, unemployed status, and being sacked from jobs, negatively influence depression symptoms. These often make the adaption process of psychological disorders to be longer and harder [53]. The experience of being diagnosed with cancer could become a source of distress in addition to the disruption in the work role. Failure to remain in employment could deprive an individual from social contact and well-being.

Patients who had colon, prostate and cervical cancer were less likely to have depression compared with those with breast cancer. This finding was similar in other studies [54]. The possible explanation for this occurrence might be increased worriedness of patients after breast cancer diagnosis, to shortened survival time, recurrence and metastasis. Furthermore, female patients face impaired body image and decreased quality of sexual life caused by surgery and other treatments. Therefore, compared with other malignant tumors, the incidence of depression and other psychiatric symptoms in breast cancer patients are higher, which is also closely related to the lack of female secondary characteristics and physical symptoms such as nausea, vomiting, fatigue, hair loss, and insomnia caused by chemotherapy [55].

Moreover, patients with stage four had 7.4 times more likely at risk of developing depression compared to patients with stage one. Similar findings have been found in other studies [14, 46]. This might be due to when the stage is advanced, patients may fear or stress. Studies found that individuals diagnosed at an advanced stage have a higher risk of developing depression compared with those diagnosed at an early stage [14, 56]. Patients with advanced-stage cancer generally have a greater need for supportive care, due to poor physical functioning, a greater symptom burden, and higher levels of distress, and depression than other cancer patients [57]. Earlier and more intensive supportive care for patients with cancer reduces symptom burden and may prolong life for patients with advanced disease [58]. For managing their distress, pharmacotherapy and psychotherapy are effective for managing depression among advanced cancer patients [59].

The preventive strategies toward the risk factors of depression include education and counselling programs that must be designed and planned according to the patient’s wishes and learning needs [60]. The current study is conducted in light with some limitations. First, some of the depression symptoms may have been due to the cancer itself or its treatment rather than due to depression.

## Conclusion

Depression is found to be a major public health problem for adult cancer patients in Ethiopia. Those cancer patients who had unemployed status, sacked from jobs, and stage IV cancer were most likely to develop depression, whereas patients having completed primary education, colon, prostate and cervical cancer were less likely to have depression. To reduce the prevalence of depression among cancer patients, designing strategies and special attention are needed to bring changes in the psychological status of patients in advanced disease stage.

## Supporting information

S1 Text Sociodemographic questions. (DOCX)

S2 Text Clinical and behavioral characteristics of questions. (DOCX)

S1 Table Psychosocial questions. (DOCX)

S2 Table Socila support questions. (DOCX)

S3 Table Tools used to assess depression. (DOCX)

S1 File Data collection tool. (DOCX)

## Abbreviation

AOR: Adjusted Odds Ratio
CI: Confidence Interval
COR: Crud Odds ratio
PHQ: Patient Health Questionnaire
SD: Standard Deviation
SPSS: Statistical Package for Social Sciences
TASH: Tikur Anbessa Specialized Hospital

## References

[1] Sung H, Ferlay J, Siegel RL, Laversanne M, Soerjomataram I, Jemal A, et al. Global cancer statistics 2020: GLOBOCAN estimates of incidence and mortality worldwide for 36 cancers in 185 countries. CA: a cancer journal for clinicians. 2021;71(3):209–49. 33538338 10.3322/caac.21660

[2] White MC, Holman DM, Boehm JE, Peipins LA, Grossman M, Jane Henley S. Age and Cancer Risk: A Potentially Modifiable Relationship. American Journal of Preventive Medicine. 2014;46(3). doi: 10.1016/j.amepre.2013.10.029 24512933 PMC4544764

[3] Avgerinos KI, Spyrou N, Mantzoros CS, Dalamaga M. Obesity and cancer risk: Emerging biological mechanisms and perspectives. Metabolism. 2019;92:121–35. doi: 10.1016/j.metabol.2018.11.001 30445141

[4] Key TJ, Allen NE, Spencer EA, Travis RC. The effect of diet on risk of cancer. The Lancet. 2002;360(9336):861–8. doi: 10.1016/S0140-6736(02)09958-0 12243933

[5] Seitz HK, Becker P. Alcohol metabolism and cancer risk. Alcohol Res Health. 2007;30(1):38–47. 17718399 PMC3860434

[6] Rasheed S, Rehman K, Akash MSH. An insight into the risk factors of brain tumors and their therapeutic interventions. Biomedicine & Pharmacotherapy. 2021;143. doi: 10.1016/j.biopha.2021.112119 34474351

[7] Pandey M, Sarita GP, Devi N, Thomas BC, Hussain BM, Krishnan R. Distress, anxiety, and depression in cancer patients undergoing chemotherapy. World Journal of Surgical Oncology. 2006;4(1):68. 17002797 10.1186/1477-7819-4-68 PMC1592486

[8] Chohan TA, Qayyum A, Rehman K, Tariq M, Akash MSH. An insight into the emerging role of cyclin-dependent kinase inhibitors as potential therapeutic agents for the treatment of advanced cancers. Biomedicine & Pharmacotherapy. 2018;107:1326–41. doi: 10.1016/j.biopha.2018.08.116 30257348

[9] Nygren P. What is cancer chemotherapy? Acta Oncologica. 2001;40(2–3):166–74. doi: 10.1080/02841860151116204 11441929

[10] Ng CG, Boks MPM, Zainal NZ, de Wit NJ. The prevalence and pharmacotherapy of depression in cancer patients. Journal of Affective Disorders. 2011;131(1):1–7. doi: 10.1016/j.jad.2010.07.034 20732716

[11] Polikandrioti M, Evaggelou E, Zerva S, Zerdila M, Koukoularis D, Kyritsi E. Evaluation of depression in patients undergoing chemotherapy. Health Science Journal. 2008;2(3).

[12] Prieto JM, Blanch J, Atala J, Carreras E, Rovira M, Cirera E, et al. Psychiatric morbidity and impact on hospital length of stay among hematologic cancer patients receiving stem-cell transplantation. Journal of Clinical Oncology. 2002;20(7):1907–17. doi: 10.1200/JCO.2002.07.101 11919251

[13] So WK, Marsh G, Ling WM, Leung FY, Lo JC, Yeung M, et al. Anxiety, depression and quality of life among Chinese breast cancer patients during adjuvant therapy. European journal of oncology nursing: the official journal of European Oncology Nursing Society. 2010;14(1):17–22. doi: 10.1016/j.ejon.2009.07.005 19734087

[14] Weiss Wiesel TR, Nelson CJ, Tew WP, Hardt M, Mohile SG, Owusu C, et al. The relationship between age, anxiety, and depression in older adults with cancer. Psycho‐Oncology. 2015;24(6):712–7. doi: 10.1002/pon.3638 25099337 PMC4320028

[15] Krebber A, Buffart L, Kleijn G, Riepma I, De Bree R, Leemans C, et al. Prevalence of depression in cancer patients: a meta‐analysis of diagnostic interviews and self‐report instruments. Psycho‐Oncology. 2014;23(2):121–30. doi: 10.1002/pon.3409 24105788 PMC4282549

[16] Pinquart M, Duberstein PR. Depression and cancer mortality: a meta-analysis. Psychol Med. 2010;40(11):1797–810. doi: 10.1017/S0033291709992285 20085667 PMC2935927

[17] Satin JR. Review: depression is associated with increased cancer mortality. Evidence-based mental health. 2010;13(2):41. doi: 10.1136/ebmh.13.2.41 21856604

[18] Smith HR. Depression in cancer patients: Pathogenesis, implications and treatment. Oncology letters. 2015;9(4):1509–14. doi: 10.3892/ol.2015.2944 25788991 PMC4356432

[19] Satin JR, Linden W, Phillips MJ. Depression as a predictor of disease progression and mortality in cancer patients: a meta‐analysis. Cancer. 2009;115(22):5349–61. doi: 10.1002/cncr.24561 19753617

[20] Wang Y, Duan Z, Ma Z, Mao Y, Li X, Wilson A, et al. Epidemiology of mental health problems among patients with cancer during COVID-19 pandemic. Translational Psychiatry. 2020 10(263). doi: 10.1038/s41398-020-00950-y 32737292 PMC7393344

[21] Khalil A, Faheem M, Fahim A, Innocent H, Mansoor Z, Rizvi S, et al. Prevalence of Depression and Anxiety amongst Cancer Patients in a Hospital Setting: A Cross-Sectional Study. Psychiatry Journal. 2016;2016. 10.1155/2016/3964806 PMC5056305 27752508

[22] Clinton-McHarg T, Carey M, Sanson-Fisher R, Tzelepis F, Bryant J, Williamson A. Anxiety and depression among haematological cancer patients attending treatment centres: Prevalence and predictors. Journal of Affective Disorders 2014;165:176–81. doi: 10.1016/j.jad.2014.04.072 24882197

[23] Tsaras K, Papathanasiou IV, Mitsi D, Veneti A, Kelesi M, Zyga S, et al. Assessment of Depression and Anxiety in Breast Cancer Patients: Prevalence and Associated Factors. Asian Pac J Cancer Prev. 2018;19(6):1661–9. doi: 10.22034/APJCP.2018.19.6.1661 29938451 PMC6103579

[24] Abuelgasim KA, Ahmed GY, Alqahtani JA, Alayed AM, Alaskar AS, Malik MA. Depression and anxiety in patients with hematological malignancies, prevalence, and associated factors. Saudi Med J 2016;37(8):877–81. doi: 10.15537/smj.2016.8.14597 27464865 PMC5018705

[25] Uwayezu MG, Gishoma D, Sego R, Mukeshimana M, Collins A. Anxiety and Depression Among Cancer Patients: Prevalence and Associated Factors at a Rwandan Referral Hospital. Rwanda Journal of Medicine and Health Sciences 2019;2(2).

[26] Wondimagegnehu A, Abebe W, Abraha A, Teferra S. Depression and social support among breast cancer patients in Addis Ababa, Ethiopia. BMC cancer. 2019;19(1):836. doi: 10.1186/s12885-019-6007-4 31455282 PMC6712811

[27] Berihun F, Haile S, Abawa M, Mulatie M, Shimeka A. Prevalence and correlates of anxiety and depression among cancer patients in the University of Gondar Comprehensive Specialized Hospital, Northwest Ethiopia. Arch Depress Anxiety 2017;3(2):042–8.

[28] Vodermaier A, Linden W, MacKenzie R, Greig D, Marshall C. Disease stage predicts post-diagnosis anxiety and depression only in some types of cancer. British Journal of Cancer. 2011;105(12):1814–7. doi: 10.1038/bjc.2011.503 22095232 PMC3251893

[29] Linden W, Vodermaier A, MacKenzie R, Greig D. Anxiety and depression after cancer diagnosis: prevalence rates by cancer type, gender, and age. Journal of affective disorders. 2012;141(2–3):343–51. doi: 10.1016/j.jad.2012.03.025 22727334

[30] Nelson CJ, Weinberger MI, Balk E, Holland J, Breitbart W, Roth AJ. The chronology of distress, anxiety, and depression in older prostate cancer patients. The oncologist. 2009;14(9):891–9. doi: 10.1634/theoncologist.2009-0059 19738000 PMC2881474

[31] Nikbakhsh N, Moudi S, Abbasian S, Khafri S. Prevalence of depression and anxiety among cancer patients. Caspian J Intern Med. 2014;5(3):167–70. 25202445 PMC4143739

[32] Bhattacharyya S, Bhattacherjee S, Mandal T, Das DK. Depression in cancer patients undergoing chemotherapy in a tertiary care hospital of North Bengal, India. Indian Journal of Public Health. 2017;61(1):14. doi: 10.4103/0019-557X.200252 28218157

[33] Naser AY, Hameed AN, Mustafa N, Alwafi H, Dahmash EZ, Alyami HS, et al. Depression and Anxiety in Patients With Cancer: A Cross-Sectional Study. Frontiers in Psychology. 2021;12(1067). doi: 10.3389/fpsyg.2021.585534 33935849 PMC8081978

[34] Kocalevent R-D, Berg L, Beutel ME, Hinz A, Zenger M, Härter M, et al. Social support in the general population: standardization of the Oslo social support scale (OSSS-3). BMC psychology. 2018;6(1):1–8. 30016997 10.1186/s40359-018-0249-9 PMC6050647

[35] Kroenke K, Spitzer RL, Williams JB. The PHQ‐9: validity of a brief depression severity measure. Journal of general internal medicine. 2001;16(9):606–13. doi: 10.1046/j.1525-1497.2001.016009606.x 11556941 PMC1495268

[36] Löwe B, Kroenke K, Herzog W, Gräfe K. Measuring depression outcome with a brief self-report instrument: sensitivity to change of the Patient Health Questionnaire (PHQ-9). Journal of affective disorders. 2004;81(1):61–6. doi: 10.1016/S0165-0327(03)00198-8 15183601

[37] Degefa M, Dubale B, Bayouh F, Ayele B, Zewde Y. Validation of the PHQ-9 depression scale in Ethiopian cancer patients attending the oncology clinic at Tikur Anbessa specialized hospital. BMC Psychiatry. 2020;20(1):446. doi: 10.1186/s12888-020-02850-3 32912183 PMC7488004

[38] Naser AY, Hameed AN, Mustafa N, Alwafi H, Dahmash EZ, Alyami HS, et al. Depression and anxiety in patients with cancer. A cross sectiponal study. Front Psychol. 2021;12(585534). 10.3389/fpsyg.2021.585534 PMC8081978 33935849

[39] Purkayastha D, Venkateswaran C, Nayar K, Unnikrishnan U. Prevalence of depression in breast cancer patients and its association with their quality of life: A cross-sectional observational study. Indian journal of palliative care. 2017;23(3):268. doi: 10.4103/IJPC.IJPC_6_17 28827929 PMC5545951

[40] Maneeton B, Maneeton N, Mahathep P. Prevalence of depression and its correlations: a cross-sectional study in Thai cancer patients. Asian Pacific Journal of Cancer Prevention. 2012;13(5):2039–43. doi: 10.7314/apjcp.2012.13.5.2039 22901168

[41] Hartung T, Brähler E, Faller H, Härter M, Hinz A, Johansen C, et al. The risk of being depressed is significantly higher in cancer patients than in the general population: prevalence and severity of depressive symptoms across major cancer types. European Journal of Cancer. 2017;72:46–53. doi: 10.1016/j.ejca.2016.11.017 28024266

[42] Hong JS, Tian J. Prevalence of anxiety and depression and their risk factors in Chinese cancer patients. Supportive Care in Cancer. 2014;22(2):453–9. doi: 10.1007/s00520-013-1997-y 24091720

[43] Baraki AG, Tessema GM, Demeke EA. High burden of depression among cancer patients on chemotherapy in University of Gondar comprehensive hospital and Felege Hiwot referral hospital, Northwest Ethiopia. Plos one. 2020;15(8). doi: 10.1371/journal.pone.0237837 32822434 PMC7446783

[44] Jacob L, Bleicher L, Kostev K, Kalder M. Prevalence of depression, anxiety and their risk factors in German women with breast cancer in general and gynecological practices. Journal of Cancer Research and Clinical Oncology. 2016;142(2):447–52. doi: 10.1007/s00432-015-2048-5 26377737 PMC11819146

[45] Alemayehu M, Deyessa N, Medihin G, Fekadu A. A descriptive analysis of depression and pain complaints among patients with cancer in a low income country. PloS one. 2018;13(3). 10.1371/journal.pone.0193713 PMC5841758 29513716

[46] Uwayezu MG, Gishoma D, Sego R, Mukeshimana M, Collins A. Anxiety and depression among cancer patients: prevalence and associated factors at a Rwandan referral hospital. Rwanda Journal of Medicine and Health Sciences. 2019;2(2):118–25.

[47] Irwin MR. Depression and insomnia in cancer: prevalence, risk factors, and effects on cancer outcomes. Current psychiatry reports. 2013;15(11):1–9. 10.1007/s11920-013-0404-1 PMC3836364 24078066

[48] Sotelo JL, Musselman D, Nemeroff C. The biology of depression in cancer and the relationship between depression and cancer progression. International Review of Psychiatry. 2014;26(1):16–30. doi: 10.3109/09540261.2013.875891 24716498

[49] Kim YH, Choi KS, Han K, Kim HW. A psychological intervention programme for patients with breast cancer under chemotherapy and at a high risk of depression: A randomised clinical trial. Journal of Clinical Nursing. 2018;27(3–4):572–81. doi: 10.1111/jocn.13910 28557043

[50] Zahid JA, Grummedal O, Madsen MT, Gögenur I. Prevention of depression in patients with cancer: A systematic review and meta-analysis of randomized controlled trials. Journal of Psychiatric Research. 2020;120:113–23. doi: 10.1016/j.jpsychires.2019.10.009 31655426

[51] Dauchy S, Dolbeault S, Reich M. Depression in cancer patients. EJC Supplements. 2013;11(2):205. doi: 10.1016/j.ejcsup.2013.07.006 26217129 PMC4041309

[52] Friberg AS, Rask Moustsen I, Benzon Larsen S, Hartung T, Wreford Andersen E, Halgren Olsen M, et al. Educational level and the risk of depression after prostate cancer. Acta Oncologica. 2019;58(5):722–9. doi: 10.1080/0284186X.2019.1566773 30700197

[53] Li Z, Geng W, Yin J, Zhang J. Effect of one comprehensive education course to lower anxiety and depression among Chinese breast cancer patients during the postoperative radiotherapy period—one randomized clinical trial. Radiation Oncology. 2018;13(1):111. doi: 10.1186/s13014-018-1054-6 29898748 PMC6000931

[54] Alwhaibi M, Sambamoorthi U, Madhavan S, Bias T, Kelly K, Walkup J. Cancer Type and Risk of Newly Diagnosed Depression Among Elderly Medicare Beneficiaries With Incident Breast, Colorectal, and Prostate Cancers. J Natl Compr Canc Netw. 2017;15(1):46–55. doi: 10.6004/jnccn.2017.0006 28040719 PMC5527325

[55] Zhu G, Li J, Li J, Wang X, Dai M, Chen J. Depression and survival of breast cancer patients: A protocol for systematic review and meta-analysis. Medicine. 2020;99(48). doi: 10.1097/MD.0000000000023399 33235118 PMC7710216

[56] Danese M, O’malley C, Lindquist K, Gleeson M, Griffiths R. An observational study of the prevalence and incidence of comorbid conditions in older women with breast cancer. Annals of Oncology. 2012;23(7):1756–65. doi: 10.1093/annonc/mdr486 22039090 PMC3387819

[57] Quist M, Adamsen L, Rørth M, Laursen JH, Christensen KB, Langer SW. The impact of a multidimensional exercise intervention on physical and functional capacity, anxiety, and depression in patients with advanced-stage lung cancer undergoing chemotherapy. Integrative cancer therapies. 2015;14(4):341–9. doi: 10.1177/1534735415572887 25800229

[58] Rosenstein DL. Depression and end-of-life care for patients with cancer. Dialogues in clinical neuroscience. 2022. 10.31887/DCNS.2011.13.1/drosenstein PMC3181973 21485750

[59] Akechi T. Psychotherapy for Depression Among Patients with Advanced Cancer. Japanese Journal of Clinical Oncology. 2012;42(12):1113–9. doi: 10.1093/jjco/hys152 23018580

[60] Djurdjević A, Nikolić S. Education of cancer patients—a psychosocial support in the holistic anticancer treatment. J BUON. 2006;11(2):217–21. 17318974

