# Supplementary material for: Prevalence of depression and associated factors among adult cancer patients receiving chemotherapy during the era of COVID-19 in Ethiopia. Hospital-based cross-sectional study
Source: PLoS One. 2022 Jun 24;17(6):e0270293. doi: 10.1371/journal.pone.0270293 (PMC9232136; doi:10.1371/journal.pone.0270293)
Supplement: S1 Text — (DOCX) [file pone.0270293.s001.docx]

Part-1 Socio demographic questions.

Code number: _______________

| No | Variables | Responses |
| --- | --- | --- |
|  | Sex | 1. Male 2. Female |
|  | Age in years |  |
|  | Educational level | 1. No formal education 2. Primary education completed 3. Primary education completed 4. Secondary School completed 5. College/ University |
|  | Marital Status | - - - 1. Single 3. Married       2. Widowed 4. Divorced |
| 5 | Occupation | 1. Civil servant 2. House wife 3. Private work 4. Others |
|  | Average monthly income in ETB |  |
