# Supplementary material for: Prevalence of depression and associated factors among adult cancer patients receiving chemotherapy during the era of COVID-19 in Ethiopia. Hospital-based cross-sectional study
Source: PLoS One. 2022 Jun 24;17(6):e0270293. doi: 10.1371/journal.pone.0270293 (PMC9232136; doi:10.1371/journal.pone.0270293)
Supplement: S2 Text — (DOCX) [file pone.0270293.s002.docx]

**Part II: Clinical characteristics**

| S.no | **Items** | **Response** |  |  |
| --- | --- | --- | --- | --- |
| 1 | Type of cancer |  |  |  |
| 2 | Stage of cancer | - - - 1. Stage I       2. Stage II       3. Stage III       4. Stage IV |  |  |
| 3 | Duration of the disease in months |  |  |  |
| 4 | Duration of chemotherapy in moths |  |  |  |
| 5 | Do you have a history of known DM? | - - - 1. Yes       2. No |  |  |
| 6 | Do you have a history of known cardiovascular disease? | 1. Yes 2. No |  |  |
| 7 | Do you have a history of known hypertension? | 1. Yes  2. No |  |  |
| 8 | Do you have a history of known k*idney* disease? | 1. Yes 2. No |  |  |
| 9 | Presence of comorbidities? | 1. Yes 2. No |  |  |
| 10 | Family history of with known mental health |  |  |  |
| 11 | Wight in Kg |  |  |  |
| 12 | Height in meters |  |  |  |
| 13 | BMI |  |  |  |
|  |  |  |  |  |
|  | **Substance Use** |  |  |  |
|  | Questions | Responses |  |  |
| 1 | khat | 1.Yes  2.Quitted  3.Never used at all |  |  |
| 2 | Tobacco products (cigarettes, chewing tobacco) | 1. Yes  2. Quitted  3. Never used at all |  |  |
| 3 | Alcoholic beverages (beer, wine, Tela, Areke etc.)? | 1. Yes  2. Quitted  3. Never drink at all |  |  |
|  |  |  |  |  |
|  |  |  |  |  |
