# Supplementary material for: Prevalence of depression and associated factors among adult cancer patients receiving chemotherapy during the era of COVID-19 in Ethiopia. Hospital-based cross-sectional study
Source: PLoS One. 2022 Jun 24;17(6):e0270293. doi: 10.1371/journal.pone.0270293 (PMC9232136; doi:10.1371/journal.pone.0270293)
Supplement: S1 Table — (DOCX) [file pone.0270293.s003.docx]

| **Part III: Psychosocial factors/life threatening events** | | | |
| --- | --- | --- | --- |
| ***Health risk*** | | | |
| Q No. | Questionnaires | Responses |  |
| 1 | In the last 6 months, have you yourself suffered a serious illness, injury or an assault? | 1`.Yes  2.No |  |
| 2 | In the last 6 months has a serious illness, injury or assault happened to a close relative? | 1.Yes  2.No |  |
| **Loss of loved ones** | | | |
| 3 | In the last 6 months has your spouse, parent or child died? | 1. Yes  2. No |  |
| 4 | In the last 6 months has a close family friend or another relative died? | 1.Yes  2. No |  |
| ***Financial stress*** | | | |
| 5 | In the last 6 months have you had a major financial crisis (serious money worries)? | 1.Yes  2.No |  |
| 6 | In the last 6 months, have you been sacked from job? | 1.Yes  2.No |  |
| 7 | In the last 6 months have you been unemployed? Not been able to work | 1.Yes  2.No |  |
| ***Relationship problem*** | | | |
| 8 | In the last 6 months have you had a separation due to marital difficulties? | 1.Yes  2.No |  |
| 9 | In the last 6 months have you broken off a steady friendship or relationship? | 1.Yes  2.No |  |
| 10 | In the last 6 months have you had a serious problem with a close friend, neighbor or relative? | 1.Yes  2.No |  |
| ***Legal issues*** | | | |
| 11 | In the last 6 months have you lost or had anything stolen which mattered a lot to you? | 1. Yes  2. No |  |
| 12 | In the last 6 months, have you had any problems with the police or courts? | 1.Yes  2.No |  |
