# Supplementary material for: Prevalence of depression and associated factors among adult cancer patients receiving chemotherapy during the era of COVID-19 in Ethiopia. Hospital-based cross-sectional study
Source: PLoS One. 2022 Jun 24;17(6):e0270293. doi: 10.1371/journal.pone.0270293 (PMC9232136; doi:10.1371/journal.pone.0270293)
Supplement: S2 Table — (DOCX) [file pone.0270293.s004.docx]

**Part VI: Questions on social support**

| No | Exposure variable | Alternative Responses |
| --- | --- | --- |
| 20 | How many people are you so close to that you can count on them if you have great personal problems? | - - - 1. None       2. 1-2       3. 3-5       4. 5+ |
| 21 | How much interest and concern do people show in what you do? | 1.None  2. Little  3. Uncertain  4. Some  5. A lot |
| 22 | How easy is it to get practical help from neighbors if you should need it? | 1. Very difficult 2. Difficult 3. Easy 4. Very easy |
| 23 | How do you explain the support of your husband during cancer? | 1.Poor  2.Moderate  3. Strong |
| 24 | Do you get practical support from family members during this disease? | 1. 1. Yes 2. 2. No |
