# Supplementary material for: Prevalence of depression and associated factors among adult cancer patients receiving chemotherapy during the era of COVID-19 in Ethiopia. Hospital-based cross-sectional study
Source: PLoS One. 2022 Jun 24;17(6):e0270293. doi: 10.1371/journal.pone.0270293 (PMC9232136; doi:10.1371/journal.pone.0270293)
Supplement: S3 Table — (DOCX) [file pone.0270293.s005.docx]

**PHQ-9 Patient Depression Questionnaire**

| Over the last 2 weeks, how often have you been bothered by any of the following problems? | Not at all | Several days | More than half the days | Nearly every day |
| --- | --- | --- | --- | --- |
| 1. Little interest or pleasure in doing things |  |  |  |  |
| 2. Feeling down, depressed, or hopeless |  |  |  |  |
| 3. Trouble falling or staying asleep, or sleeping too much |  |  |  |  |
| 4. Feeling tired or having little energy |  |  |  |  |
| 5. Poor appetite or overeating |  |  |  |  |
| 6. Feeling bad about yourself or that you are a failure or have let yourself or your family down |  |  |  |  |
| 7. Trouble concentrating on things, such as reading the newspaper or watching television |  |  |  |  |
| 8. Moving or speaking so slowly that other people could have noticed. Or the opposite being so figety or restless that you have been moving around a lot more than usual |  |  |  |  |
| 9. Thoughts that you would be better off dead, or of hurting yourself |  |  |  |  |
| Add column |  |  |  |  |
| Total score |  |  |  |  |
