# Supplementary material for: Prevalence of depression and associated factors among adult cancer patients receiving chemotherapy during the era of COVID-19 in Ethiopia. Hospital-based cross-sectional study
Source: PLoS One. 2022 Jun 24;17(6):e0270293. doi: 10.1371/journal.pone.0270293 (PMC9232136; doi:10.1371/journal.pone.0270293)
Supplement: S1 File — (DOCX) [file pone.0270293.s006.docx]

**S1 file: Data collection tool**

**Table 1. Socio-demographic questions**.

Code number: _______________

| S.No. | Variables | Responses |
| --- | --- | --- |
| 1 | Sex | 1. Male 2. Female |
| 2 | Age in years |  |
| 3 | Educational level | 1. No formal education 2. Primary education completed 3. Primary education completed 4. Secondary School completed 5. College/ University |
| 4 | Marital Status | - - - 1. Single 3. Married       2. Widowed 4. Divorced |
| 5 | Occupation | 1. Civil servant 2. House wife 3. Private work 4. Others |
| 6 | Average monthly income in Ethiopia Birr (ETB) |  |

**Table 2. Clinical characteristics**

| S. No. | Items | Response |
| --- | --- | --- |
| 1 | Type of cancer |  |
| 2 | Stage of cancer | - - - 1. Stage I       2. Stage II       3. Stage III       4. Stage IV |
| 3 | Duration of the disease in months |  |
| 4 | Duration of chemotherapy in moths |  |
| 5 | Do you have a history of known DM? | - - - 1. Yes       2. No |
| 6 | Do you have a history of known cardiovascular disease? | 1. Yes 2. No |
| 7 | Do you have a history of known *h*ypertension? | 1. Yes 2. No |
| 8 | Do you have a history of known k*idney* disease? | 1. Yes 2. No |
| 9 | Presence of comorbidities? | 1. Yes 2. No |
| 10 | Family history of with known mental health | 1. Yes 2. No |
| 11 | Wight in Kg |  |
| 12 | Height in meters |  |
| 13 | BMI |  |
|  | **Substance Use** |  |
|  | Questions | Responses |
| 1 | khat | 1.Yes  2.Quitted  3.Never used at all |
| 2 | Tobacco products (cigarettes, chewing tobacco) | 1. Yes  2. Quitted  3. Never used at all |
| 3 | Alcoholic beverages (beer, wine, Tela, Areke etc.)? | 1. Yes 2. Quitted 3. Never drink at all |

| ***Health risk*** | | |
| --- | --- | --- |
| Q No. | Questionnaires | Responses |
| 1 | In the last 6 months, have you yourself suffered a serious illness, injury or an assault? | 1`.Yes  2.No |
| 2 | In the last 6 months has a serious illness, injury or assault happened to a close relative? | 1.Yes  2.No |
| ***Loss of loved ones*** | | |
| 3 | In the last 6 months has your spouse, parent or child died? | 1. Yes  2. No |
| 4 | In the last 6 months has a close family friend or another relative died? | 1.Yes  2. No |
| ***Financial stress*** | | |
| 5 | In the last 6 months have you had a major financial crisis (serious money worries)? | 1.Yes  2.No |
| 6 | In the last 6 months, have you been sacked from job? | 1.Yes  2.No |
| 7 | In the last 6 months have you been unemployed? Not been able to work | 1.Yes  2.No |
| ***Relationship problem*** | | |
| 8 | In the last 6 months have you had a separation due to marital difficulties? | 1.Yes  2.No |
| 9 | In the last 6 months have you broken off a steady friendship or relationship? | 1.Yes  2.No |
| 10 | In the last 6 months have you had a serious problem with a close friend, neighbor or relative? | 1.Yes  2.No |
| ***Legal issues*** | | |
| 11 | In the last 6 months have you lost or had anything stolen which mattered a lot to you? | 1. Yes  2. No |
| 12 | In the last 6 months, have you had any problems with the police or courts? | 1.Yes  2.No |

**Table 3. Psychosocial factors/life threatening events**

**Table 4. Questions on social support**

| S.No. | Exposure variable | Alternative Responses |
| --- | --- | --- |
| 20 | How many people are you so close to that you can count on them if you have great personal problems? | - - - 1. None       2. 1-2       3. 3-5       4. 5+ |
| 21 | How much interest and concern do people show in what you do? | 1. None 2. Little 3. Uncertain 4. Some 5. A lot |
| 22 | How easy is it to get practical help from neighbors if you should need it? | 1. Very difficult 2. Difficult 3. Easy 4. Very easy |
| 23 | How do you explain the support of your husband during cancer? | 1. Poor 2. Moderate 3. Strong |
| 24 | Do you get practical support from family members during this disease? | 1. Yes 2. No |

**Table 5. PHQ-9 Patient Depression Questionnaire**

| Over the last 2 weeks, how often have you been bothered by any of the following problems? | Not at all | Several days | More than half the days | Nearly every day |
| --- | --- | --- | --- | --- |
| 1. Little interest or pleasure in doing things |  |  |  |  |
| 1. Feeling down, depressed, or hopeless |  |  |  |  |
| 1. Trouble falling or staying asleep, or sleeping too much |  |  |  |  |
| 1. Feeling tired or having little energy |  |  |  |  |
| 1. Poor appetite or overeating |  |  |  |  |
| 1. Feeling bad about yourself or that you are a failure or have let yourself or your family down |  |  |  |  |
| 1. Trouble concentrating on things, such as reading the newspaper or watching television |  |  |  |  |
| 1. Moving or speaking so slowly that other people could have noticed. Or the opposite being so figety or restless that you have been moving around a lot more than usual |  |  |  |  |
| 1. Thoughts that you would be better off dead, or of hurting yourself |  |  |  |  |
| Add column |  |  |  |  |
| Total score |  |  |  |  |
