# Supplementary material for: Prevalence of depression and associated factors among adult cancer patients receiving chemotherapy during the era of COVID-19 in Ethiopia. Hospital-based cross-sectional study
Source: PLoS One. 2022 Jun 24;17(6):e0270293. doi: 10.1371/journal.pone.0270293 (PMC9232136; doi:10.1371/journal.pone.0270293)
Supplement: Attachment — Submitted filename: Response to reveiwer.docx [file pone.0270293.s007.docx]

**Author response for comments**

PLOS ONE

PONE-D-22-06018

Prevalence of depression and associated factors among adult cancer patients receiving chemotherapy during the era of COVID-19 in Ethiopia. A Hospital based cross sectional study

PLOSONE

Dear Dr. Belete,

Thank you for submitting your manuscript to PLOS ONE. After careful consideration, we feel that it has merit but does not fully meet PLOS ONE’s publication criteria as it currently stands. Therefore, we invite you to submit a revised version of the manuscript that addresses the points raised during the review process.

Response: Dear editor, thank you very much for allowing us to revise our manuscript. We have tried to respond to each concern as much as possible. We have submitted a revised version of the manuscript.

I have received the reports from our advisors on your manuscript which you submitted to PLOS ONE.

Based on the comments received, I feel that your manuscript could be reconsidered for publication should you be prepared to incorporate major revisions. When preparing your revised manuscript, you are asked to carefully consider the reviewer comments below and submit a list of responses to the comments.

Author response: Dear editor, thank you for considering our manuscript publication. We have addressed the points raised by the reviewer and editors.

Editor Comments: The paper should be checked by a professional speaker of English before complete acceptance.

Author response: Dear editor, thank you very much. We have tried to edit the manuscript and also we used the online Grammarlly using the following links: https://www.grammarly.com/

Please submit your revised manuscript by May 19 2022 11:59 PM. If you will need more time than this to complete your revisions, please reply to this message or contact the journal office at [plosone@plos.org](mailto:plosone@plos.org). When you're ready to submit your revision, log on to <https://www.editorialmanager.com/pone/> and select the 'Submissions Needing Revision' folder to locate your manuscript file.

Author response: Dear editor, thank you. We have submitted per the due date.

Please include the following items when submitting your revised manuscript:

- A rebuttal letter that responds to each point raised by the academic editor and reviewer(s). You should upload this letter as a separate file labeled 'Response to Reviewers'.
- A marked-up copy of your manuscript that highlights changes made to the original version. You should upload this as a separate file labeled 'Revised Manuscript with Track Changes'.
- An unmarked version of your revised paper without tracked changes. You should upload this as a separate file labeled 'Manuscript'.

Author response: Dear editor, thank you. We have included ‘Response to Reviewers', 'Revised Manuscript with Track Changes’, and ‘Manuscript’ separately.

If you would like to make changes to your financial disclosure, please include your updated statement in your cover letter. Guidelines for resubmitting your figure files are available below the reviewer comments at the end of this letter.

Author response: Dear editor, thanks for allowing us to update the financial disclosure. We have added it to the cover letter. Thanks for the suggestion for guidelines for figures.

If applicable, we recommend that you deposit your laboratory protocols in [protocols.io](http://protocols.io) to enhance the reproducibility of your results. Protocols.io assigns your protocol its own identifier (DOI) so that it can be cited independently in the future. For instructions see: <https://journals.plos.org/plosone/s/submission-guidelines#loc-laboratory-protocols>. Additionally, PLOS ONE offers an option for publishing peer-reviewed Lab Protocol articles, which describe protocols hosted on [protocols.io](http://protocols.io). Read more information on sharing protocols at [https://plos.org/protocols?utm_medium=editorial email&utm_source=authorletters&utm_campaign=protocols](https://plos.org/protocols?utm_medium=editorial%20email&utm_source=authorletters&utm_campaign=protocols).
Author response: Dear editor, thank you for recommending depositing the laboratory protocols in protocols.io. But it is not applicable
We look forward to receiving your revised manuscript.

Kind regards,

Muhammad Sajid Hamid Akash

Academic Editor PLOS ONE

Journal Requirements:

When submitting your revision, we need you to address these additional requirements.

1. Please ensure that your manuscript meets PLOS ONE's style requirements, including those for file naming. The PLOS ONE style templates can be found at

<https://journals.plos.org/plosone/s/file?id=wjVg/PLOSOne_formatting_sample_main_body.pdf> and

<https://journals.plos.org/plosone/s/file?id=ba62/PLOSOne_formatting_sample_title_authors_affiliations.pdf>

Author response: Dear Editor, we saw the link you invited me and I made corrections to the manuscript style to meet the requirement of PLOS ONE.

2. We suggest you thoroughly copyedit your manuscript for language usage, spelling, and grammar. If you do not know anyone who can help you do this, you may wish to consider employing a professional scientific editing service.

Whilst you may use any professional scientific editing service of your choice, PLOS has partnered with both American Journal Experts (AJE) and Editage to provide discounted services to PLOS authors. Both organizations have experience helping authors meet PLOS guidelines and can provide language editing, translation, manuscript formatting, and figure formatting to ensure your manuscript meets our submission guidelines. To take advantage of our partnership with AJE, visit the AJE website (<http://learn.aje.com/plos/>) for a 15% discount off AJE services. To take advantage of our partnership with Editage, visit the Editage website ([www.editage.com](http://www.editage.com)) and enter referral code PLOSEDIT for a 15% discount off Editage services.  If the PLOS editorial team finds any language issues in text that either AJE or Editage has edited, the service provider will re-edit the text for free.

Author response: Dear editor, thank you for your suggestion.

Upon resubmission, please provide the following:

The name of the colleague or the details of the professional service that edited your manuscript

Author response: Dear Editor, we edit the manuscript by using an online grammar checker.

A copy of your manuscript showing your changes by either highlighting them or using track changes (uploaded as a *supporting information* file)

Author response: Dear Editor, we have included manuscripts that contain track changes and uploaded them as manuscript track changes.

A clean copy of the edited manuscript (uploaded as the new *manuscript* file)”

Author response: Dear Editor, thank you. We have included a clean copy of the edited manuscript and uploaded them as a manuscript file.

3. Please provide additional details regarding participant consent. In the ethics statement in the Methods and online submission information, please ensure that you have specified (1) whether consent was informed and (2) what type you obtained (for instance, written or verbal, and if verbal, how it was documented and witnessed). If your study included minors, state whether you obtained consent from parents or guardians. If the need for consent was waived by the ethics committee, please include this information.

If you are reporting a retrospective study of medical records or archived samples, please ensure that you have discussed whether all data were fully anonymized before you accessed them and/or whether the IRB or ethics committee waived the requirement for informed consent. If patients provided informed written consent to have data from their medical records used in research, please include this information.

Author response: Dear Editor, 1) consent was informed 2) the consent obtained was verbal informed consent. Detail of the study’s objective, method of data collection, and ethical concern were explained to potential participants, and they were requested to participate in the study voluntarily. For volunteer participants, data collectors tick the right sign in front of I agree, then continue interviewing the participant. There were no minors in this study, all were adults who can themselves give consent after understanding what was explained about the study. The study participants gave verbal consent to use their medical cards.

4. Thank you for stating the following financial disclosure:

"The funders had no role in study design, data collection and analysis, decision to publish, or preparation of the manuscript."

At this time, please address the following queries:

a) Please clarify the sources of funding (financial or material support) for your study. List the grants or organizations that supported your study, including funding received from your institution.

Author response: Dear Editor, sorry for making confused. It is a technical error this work had not received any form of support. All cost is covered by author.

State what role the funders took in the study. If the funders had no role in your study, please state: “The funders had no role in study design, data collection and analysis, decision to publish, or preparation of the manuscript.”

Author response: Dear Editor, again sorry. This study did not receive any form of support. Therefore, there is no funder.

b) If any authors received a salary from any of your funders, please state which authors and which funders.

Author response: Dear Editor, since no funder was participated so no author is received salary from funders.

d) If you did not receive any funding for this study, please state: “The authors received no specific funding for this work.”

 Author response: Dear Editor, the authors received no specific funding for this work.

Please include your amended statements within your cover letter; we will change the online submission form on your behalf.

Author response: Dear Editor, thank you very much. The amended statement is within the cover letter.

5. PLOS requires an ORCID iD for the corresponding author in Editorial Manager on papers submitted after December 6th, 2016. Please ensure that you have an ORCID iD and that it is validated in Editorial Manager. To do this, go to ‘Update my Information’ (in the upper left-hand corner of the main menu), and click on the Fetch/Validate link next to the ORCID field. This will take you to the ORCID site and allow you to create a new iD or authenticate a pre-existing iD in Editorial Manager. Please see the following video for instructions on linking an ORCID iD to your Editorial Manager account: <https://www.youtube.com/watch?v=_xcclfuvtxQ>

[Note: HTML markup is below. Please do not edit.]

Author response: Dear Editor, thank you for your recommendation. Already I have an ORCID id

Reviewers' comments:

Reviewer's Responses to Questions

**Comments to the Author**

1. Is the manuscript technically sound, and do the data support the conclusions?

The manuscript must describe a technically sound piece of scientific research with data that supports the conclusions. Experiments must have been conducted rigorously, with appropriate controls, replication, and sample sizes. The conclusions must be drawn appropriately based on the data presented.

Reviewer #1: Partly

Author response: Dear reviewer, we have tried to modify it. See the revised track changes.

2. Has the statistical analysis been performed appropriately and rigorously?

Reviewer #1: Yes

Author response: Dear reviewer, thank you very much.

3. Have the authors made all data underlying the findings in their manuscript fully available?

The [PLOS Data policy](http://www.plosone.org/static/policies.action#sharing) requires authors to make all data underlying the findings described in their manuscript fully available without restriction, with rare exception (please refer to the Data Availability Statement in the manuscript PDF file). The data should be provided as part of the manuscript or its supporting information, or deposited to a public repository. For example, in addition to summary statistics, the data points behind means, medians and variance measures should be available. If there are restrictions on publicly sharing data—e.g. participant privacy or use of data from a third party—those must be specified.

Reviewer #1: Yes

Author response: Dear reviewer, thank you

4. Is the manuscript presented in an intelligible fashion and written in standard English?

PLOS ONE does not copyedit accepted manuscripts, so the language in submitted articles must be clear, correct, and unambiguous. Any typographical or grammatical errors should be corrected at revision, so please note any specific errors here.

Reviewer #1: Yes

Author response: Dear reviewer, thank you very much

5. Review Comments to the Author

Please use the space provided to explain your answers to the questions above. You may also include additional comments for the author, including concerns about dual publication, research ethics, or publication ethics. (Please upload your review as an attachment if it exceeds 20,000 characters)

Response: Dear editor, thank you very much. We have tried to answer the reviewer’s feedback and question as much as we can.

Reviewer #1: The author presented an original article on ‘Prevalence of depression and associated factors among adult cancer patients receiving chemotherapy during the era of COVID-19 in Ethiopia. A Hospital based cross sectional study’. The manuscript needs to be improved regarding the following aspects.
• Cancer chemotherapy should be described in detail to strengthen your research study moreover, overall associated factors for cancer risk should also be included in the introduction part, Biomed Pharmacother. 2021; 143: 112119. <https://doi.org/10.1016/j.biopha.2021.112119>

Author response: Dear reviewer, thank you for your recommendation. As per your recommendation, we have made an amendment to the revised manuscript.
• In the discussion section, discuss more details of the results and their outcome risks and preventive strategies.

Author response: Dear reviewer, thank you very much. We have tried to address all the points you raised in the revised manuscript.
• More description is required to explain the association between key points and the conclusion.

Author response: Dear reviewer, we have tried to add more description in the discussion part that links to a conclusion.
• In this article not a single graphical representation has been included, add approximately 3 to 4 figures or graphs to strengthen your research manuscript.

Author response: Dear reviewer, thank you very much for your effort to see each part of our work. According to your suggestion, we add 3 figures to the revised manuscript.
• There are a few formatting mistakes, for this deeply review the article.

Author response: Dear reviewer, we review deeply and modification was done in the revised manuscript.
• Many references are missing or inconsistent in format.

Author response: Dear reviewer, I made corrections as per your comment.
• There are some grammatical mistakes in the manuscript, such as verbs and prepositions. The manuscript needs extensive review by an author.

Author response: Dear reviewer, as per your comment I revise the manuscript for grammatical and spelling errors. The corrections are highlighted in the document entitled “revised manuscript with track changes”.

6. PLOS authors have the option to publish the peer review history of their article ([what does this mean?](https://journals.plos.org/plosone/s/editorial-and-peer-review-process#loc-peer-review-history)). If published, this will include your full peer review and any attached files.

If you choose “no”, your identity will remain anonymous but your review may still be made public.

**Do you want your identity to be public for this peer review?** For information about this choice, including consent withdrawal, please see our [Privacy Policy](https://www.plos.org/privacy-policy).

Reviewer #1: No

 [NOTE: If reviewer comments were submitted as an attachment file, they will be attached to this email and accessible via the submission site. Please log into your account, locate the manuscript record, and check for the action link "View Attachments". If this link does not appear, there are no attachment files.]

While revising your submission, please upload your figure files to the Preflight Analysis and Conversion Engine (PACE) digital diagnostic tool, <https://pacev2.apexcovantage.com/>. PACE helps ensure that figures meet PLOS requirements. To use PACE, you must first register as a user. Registration is free. Then, login and navigate to the UPLOAD tab, where you will find detailed instructions on how to use the tool. If you encounter any issues or have any questions when using PACE, please email PLOS at [figures@plos.org](mailto:figures@plos.org). Please note that Supporting Information files do not need this step.

Author response: Dear editor, thank you for your suggestion. We used the link that you invite me.
